# The Landscape of Somatic Copy Number Alterations in Head and Neck Squamous Cell Carcinoma

**DOI:** 10.3389/fonc.2020.00321

**Published:** 2020-03-12

**Authors:** Jian Yang, Yi Chen, Hong Luo, Haoyang Cai

**Affiliations:** ^1^Center of Growth, Metabolism, and Aging, Key Laboratory of Bio-Resources and Eco-Environment, College of Life Sciences, Sichuan University, Chengdu, China; ^2^Department of Gastrointestinal Surgery, West China Hospital, Sichuan University, Chengdu, China

**Keywords:** head and neck squamous cell carcinoma, meta-analysis, copy number alteration, chromothripsis, genomic array

## Abstract

Head and neck squamous cell carcinoma (HNSCC) is the sixth most common malignancy worldwide. Somatic copy number alterations (CNAs) play a significant role in the development of this lethal cancer. In this study, we present a meta-analysis of CNAs for a total of 1,395 HNSCC samples. Publicly available R packages and in-house scripts were used for genomic array data processing, including normalization, segmentation and CNA calling. We detected 125 regions of significant gains or losses using GISTIC algorithm and found several potential driver genes in these regions. The incidence of chromothripsis in HNSCC was estimated to be 6%, and the chromosome pulverization hotspot regions were detected. We determined 323 genomic locations significantly enriched for breakpoints, which indicate HNSCC-specific genomic instability regions. Unsupervised clustering of genome-wide CNA data revealed a sub-cluster predominantly composed of nasopharynx tumors and presented a large proportion of HPV-positive samples. These results will facilitate the discovery of therapeutic candidates and extend our molecular understanding of HNSCC.

## Introduction

Head and neck squamous cell carcinoma (HNSCC) has an incidence of over 600,000 new diagnoses per year worldwide, with a mortality rate of nearly 50% (1, 2). It consists of a heterogeneous group of epithelial tumors arising from oral cavity, nasal cavity, pharynx, larynx, paranasal sinuses and salivary glands. Tobacco use and excessive alcohol consumption are well-established major risk factors for the development of HNSCC. Recently, human papillomavirus (HPV) infection has also been recognized as an increasingly important risk factor for HNSCC (3, 4). Previous studies have concluded that many genes are recurrently mutated in HNSCC but at low frequencies, and functional consequences of these mutations are often unclear. Therefore, a more detailed understanding of the genetic mechanisms of HNSCC is needed to improve prevention and treatment of this cancer.

Genomic instability in the form of copy number alteration (CNA) is a hallmark of cancer cells and a promotor for carcinogenesis (5–8). CNAs have critical roles in activating oncogenes and in inactivating tumor suppressors, and often affect many genes simultaneously (9–13). Like many other cancer types, HNSCCs are characterized by complex patterns of copy number gains and losses throughout the genome (14–17). In 2015, The Cancer Genome Atlas (TCGA) consortium published a robust integrative multiplatform characterization of 279 HNSCCs, including genome-wide profiling of copy number alterations (18). Although many studies have identified multiple regions of CNAs in HNSCC, a comprehensive landscape of copy number changes still remains largely unexplored. The role and implications of these aberrations in HNSCC need to be further evaluated and elucidated.

Recently, a new phenomenon, called chromothripsis, has been described as a new mechanism for cancer initiation and progression (19). It is characterized by the shattering of one or multiple chromosomes followed by a random reassembly of the DNA fragments. All the events are believed to occur in a single catastrophic event rather than a series of subsequent alterations (20–23). This is in contrast to the multistep model of cancer development. It can lead to the simultaneous acquisition of multiple tumor-promoting lesions. Chromothripsis has attracted a great attention in cancer research and has been observed in many tumor types (24–27). However, a comprehensive evaluation of chromothripsis in HNSCC has not been carried out yet. Given the low incidence of chromothripsis, a large number of tumor samples are required to perform such analysis.

Here we present a meta-analysis of genomic copy number alterations for 1,395 HNSCC samples from 19 datasets. In brief, we identified 72 and 53 regions of amplification and deletion, respectively, and detected potential driver genes in these regions. We identified 92 samples with the signs of chromothripsis, giving the incidence of 6%. Furthermore, we found some chromosome pulverization hotspots in HNSCC. The hierarchical clustering analysis identified two major clusters and cluster specific CNA patterns were provided. Our analysis may facilitate further studies on the distinct molecular mechanisms underlying HNSCC.

## Materials and Methods

### Data Collection and Patient Characteristics

We collected genomic array data from NCBI Gene Expression Omnibus (GEO) (28) and TCGA (29) databases. Our data selection criteria are that (1) the patient was diagnosed with head and neck squamous cell carcinoma, excluding precancerous lesions, metastasis, and recurrence cases, (2) the array platform must be genome-wide, including array comparative genomic hybridization (aCGH) and single nucleotide polymorphisms (SNP) array, and (3) the number of probes of the array platform should be >100K.

Our cohort consists of 873 patients from GEO and 522 patients from TCGA datasets. The primary tumors originated from several sites of the head and neck region, including oral cavity (*n* = 768, 61%), nasopharynx (*n* = 73, 5.8%), oropharynx (*n* = 123, 9.7%), hypopharynx (*n* = 78, 6.2%), larynx (*n* = 186, 14.8%), and sinonasal cavity (*n* = 31, 2.5%). The majority of patients were tobacco users (69.4%), while 55.6% were users of alcohol. Approximately 14% (150 out of 1,065 patients with available HPV test results) of patients were HPV-positive which is consistent with the reported frequency at about 14.17% (85,000 in 600,000) (30). A summary of patients' clinical data is provided in Table 1.

**Table 1:** Summary of clinical data.

**Characteristic**	**Number of patients (*N* = 1,395)**	**Proportion of none missing values (%)**
Age (years)		
Range	19–93	
Mean	60.33	
Gender (n)		
Male	888	73.4
Female	322	26.6
Unknown	185	
Site of primary tumor (n)		
Oral cavity	768	61
Nasopharynx	73	5.8
Oropharynx	123	9.7
Hypopharynx	78	6.2
Larynx	186	14.8
Sinonasal	31	2.5
Unknown	136	
Tumor grade (n)		
Well differentiated	186	19
Moderately differentiated	555	56.8
Poorly differentiated	236	24.2
Unknown	418	
HPV (n)		
Yes	150	14.1
No	915	85.9
Unknown	330	
Alcohol (n)		
Yes	370	55.6
No	296	44.4
Unknown	729	
Tobacco (n)		
Yes	763	69.4
No	336	30.6
Unknown	296	

For GEO datasets, the raw signal intensity files were downloaded for re-analysis (31, 32). The tumor-paired normal samples were used as reference in data analysis, if available. If one sample was hybridized on two or more array platforms, the sample analyzed by higher resolution array was included in the following analysis. A total of 18 GEO series consist of 873 samples were collected. Supplementary Table 1 shows details of the 18 GEO series. For each patient, clinical data was extracted from GEO website, publication's main text and supplementary file, and was converted to a standardized format. For TCGA dataset, segmented genomic array data (level 3) and clinical information were downloaded from TCGA data portal. There were 522 HNSCC samples, all analyzed by Affymetrix SNP6 arrays. In total, we collected 1,395 HNSCC samples (Supplementary Table 2).

### Data Processing and Normalization

Affymetrix raw data files (.CEL) were re-analyzed by the R package aroma.affymetrix with the CRMAv.2 method (33). For non-Affymetrix arrays, probe-level signal intensity and annotation files were processed by in-house Perl scripts. When necessary, genomic coordinates were remapped to hg19/GRCh37 using UCSC's liftOver utility (34). The circular binary segmentation (CBS) (35) algorithm was employed to segment DNA copy number data. The CNA calling cut-off values for genomic gains and losses were set to 0.2 and −0.2, respectively. The X and Y chromosomes were excluded to avoid gender bias.

### GISTIC Analysis

GISTIC is a tool used for the detection of peak regions significantly amplified or deleted in a number of samples (36). Default settings were used to run GISTIC 2.0, except that (1) the false discovery rate *q*-value was set to <0.05 for peak detection, (2) the arm peel method was used to reduce noise, (3) the confidence level used to calculate the region containing a driver was set to 0.95, (4) “Extreme” method was applied for reducing marker-level to the gene-level copy number data, (5) log2 ratios >0.2 and <-0.2 were taken as thresholds for gain and loss detection. Furthermore, for known cancer-related genes, we downloaded 719 cancer consensus genes from the Catalog of Somatic Mutations in Cancer (COSMIC) database (37).

### Chromothripsis Detection

We detected chromothripsis-like phenomenon by CTLPScanner web server (http://cgma.scu.edu.cn/CTLPScanner/) (38). The segmented array data were used as input to identify clustering of copy number changes in the genome. The following parameters and thresholds were applied: copy number status switch times ≥20, log10 of Likelihood ratio ≥10, minimum segment size set to 10 Kb and signal value difference between two adjacent segments ≥0.4. For data visualization, signal value for genomic gains and losses were set to 0.2 and −0.2, respectively.

### Definition of Copy Number Alteration Breakpoint

The genomic starts and ends of copy number alterations were considered as breakpoints. We used a stringent definition of CNA breakpoints to reduce the bias caused by technical or biological noise. A genomic position was defined as breakpoint if the Log2 signal value alteration between two adjacent genomic segments was >0.4 (39, 40). Copy number segments smaller than 10 Kb were excluded from analysis. Breakpoints located in chromosomal telomeres and centromeres were ignored. To investigate the distribution of breakpoints, the subdivision of the genome and random shuffling of breakpoint positions were performed by in-house Perl scripts. The common fragile sites (CFSs) and non-fragile regions (NFR) of the human genome were obtained from previous publications (41–43). We used the liftOver tool to convert the genome coordinates from assembly hg18/NCBI36 to hg19/ GRCh37 (34).

## Results

### Genome-Wide CNA Profiles of HNSCC

**Figure 1:**
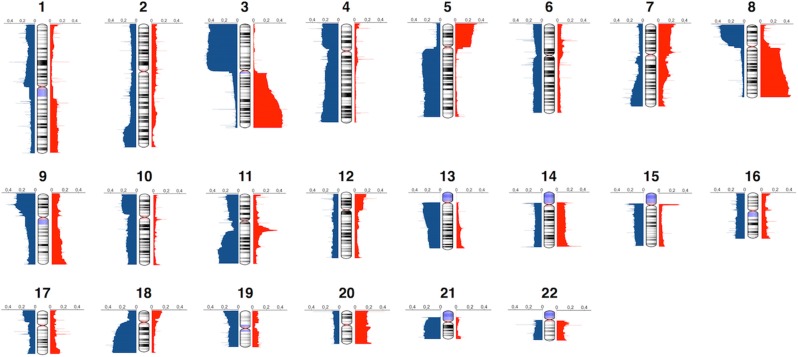
Genome-wide frequency plot of copy number alterations. Copy number gains and losses are represented in red and blue, respectively.

In our collections, the genomic alterations ranged from whole arm gains and losses to focal high-level amplifications. The mean size of the CNAs was 6.7 Mb, and the average number of CNA events per tumor sample was 443. The CNA frequency plots for each chromosome are shown in Figure 1. It provides a high-resolution view of CNA distribution across the HNSCC genome. The most commonly altered regions in HNSCC included gains of 3q, 5p, 8q, and losses of 3p, 5q, 8p, 13q, 18q, and 21q. The most notable focal CNA was the overrepresentation of 11q13, which may be associated with oncogene CCND1 amplification (44, 45). These results were consistent with previous studies (18, 46–49). We also identified several frequently altered regions that were rarely reported, such as the whole chromosome 4 loss and chromosome 20 gain.

We further investigated the frequency of arm-level alterations, which were defined as a single CNA that encompasses >50% of a chromosomal arm. Supplementary Figure 1 represents the arm-level alteration frequency measured across all samples. Similar to other cancer types, we observed a negative correlation (*r* = −0.545, *p* = 0.0003) between arm-level gain and loss frequencies (5). It reveals that frequently altered chromosomes tend to be either gained or lost, but rarely both.

### Driver Gene Identification

**Figure 2:**
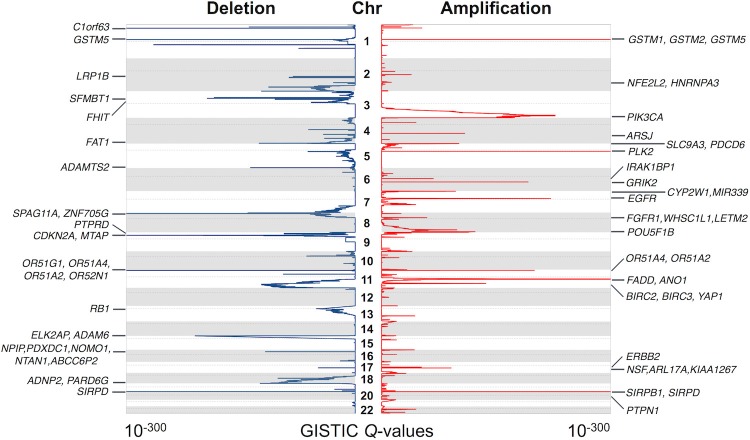
Significantly altered regions and genes identified by GISTIC algorithm. The significance of recurrent amplification and deletion is plotted across the genome. Some of the identified potential cancer driver genes are shown at the corresponding peaks.

GISTIC 2.0 (36) was performed to identify statistically significant recurrent focal CNAs and potential driver genes. We observed 72 and 53 regions of recurrent copy number gain and loss, respectively (*q* < 0.05) (Supplementary Table 3). The annotation of these regions revealed 1,160 targeted genes. Some of the most significantly altered genes and their significance levels are shown in Figure 2. Thirty-one genes were known cancer genes and listed in COSMIC database (37) (Supplementary Table 4). Among them, 17 genes have been reported to be driver genes in HNSCC, such as ASPSCR1, BIRC3, CBFA2T3, EGFR, ERBB2, FGFR1, NFE2L2, PIK3CA, RECQL4, RNF213, and WHSC1L1 were located in copy-number gain regions, while CDKN2A, FAT1, LRP1B, PTPRD, PTEN, and RB1 were identified in copy-number loss regions. In addition to these known HNSCC driver genes, we also identified novel or recently described genes. There were 39 regions of interest that contained only one candidate driver gene. IRAK1BP1 (6q14.1) is an inhibitory component of TNFR-related pathways (50, 51). Although it can activate NF-kappa-B pathway, which is known as a major regulator of innate and adaptive immune responses, it has not been found associated with cancer. PTPN1 (20q13.13) is a tyrosine-protein phosphatase and acts as a regulator of endoplasmic reticulum unfolded protein response (52, 53). It directly dephosphorylates insulin receptor and insulin receptor substrates, which results in down regulation of insulin signaling. In recent studies, the overexpression of PTPN1 was detected in several cancer types such as colon cancer and breast cancer (54, 55). It has also been reported as an indicator of poor prognosis in gastric cancer (56). These results suggest an essential role for PTPN1 in HNSCC carcinogenesis. The new candidates in these recurrent alterations may direct experimental studies and contribute to molecular mechanism researches of HNSCC.

### Chromothripsis in HNSCC

**Figure 3:**
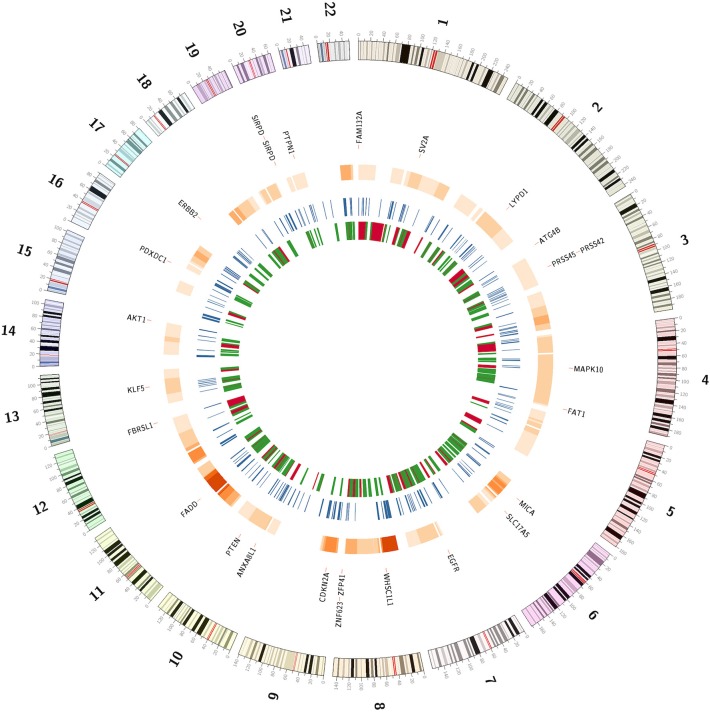
The genomic landscape of chromothripsis and copy number alteration breakpoints. The outermost circle represents potential cancer driver genes located in chromosome pulverization regions or the breakpoint hotspots. The next circle represents hotspots of chromothripsis region. The third circle shows the breakpoint-prone genomic regions. The innermost circle represents common fragile sites and non-fragile regions in red and green, respectively.

Using CTLPScanner algorithm (38), we identified 92 chromothripsis cases from 1,395 HNSCC samples, giving an incidence of about 6% (Supplementary Table 5). Compared to other tumor types, the incidence rate of HNSCC is a little bit above the average level (approximately 5% of all cancers) (12, 57, 58). The sizes of affected regions ranged from 30 Mb to the whole chromosome. Supplementary Figure 2 illustrates examples of localized and chromosome-level pulverization. In our dataset, the most frequently shattered chromosome was chromosome 8 (18%, 17 out of 92). Chromosomes 6 and 11 also exhibited a relatively high pulverization rate. We also investigated the number of affected chromosomes per tumor sample, and found that approximately 11% (10 out of 92) of chromothripsis cases carried two or more chromosome shattering events. Figure 3 shows the hotspots of chromothripsis across the genome. These results suggest that HNSCC has a specific pattern of chromothripsis. In our dataset, the follow-up data was available for 772 patients, and the mean follow-up time was 31.4 months. Chromothripsis was detected in 8.2% of this cohort. The Kaplan-Meier analysis indicated that there was no survival difference in patients with chromothripsis patterns compared with those without chromothripsis (log-rank test, *p* = 0.61) (Supplementary Figure 3).

### Copy Number Alteration Breakpoint Analysis

Chromosome breakage is a hallmark of cancer cells and may contribute to cancer initiation and progression. We identified a total of 193,028 CNA breakpoints in 1,395 HNSCC samples, with the mean breakpoints per sample being 138. We further examined the CNA breakpoint hotspots across the genome. Each chromosome was divided into continuous bins with length of 1 Mb, and the density of breakpoints per bin was calculated. Then, we shuffled the position of copy number alterations 10,000 times to obtain the background distribution of breakpoints. The breakpoint-prone genomic regions were identified through comparing the actual and background distributions. In total, we obtained 323 genomic regions that were significantly enriched for breakpoints of chromosomal rearrangements (Bonferroni corrected *p* < 0.01) (Supplementary Table 6). Moreover, we compared these regions with known common fragile sites and non-fragile regions described in the literature (41). Among these hotspots, only 58 (18%) were located within CFS, while 103 (32%) overlapped with NFS (Figure 3). These results provide important information about the HNSCC-specific genomic instability regions.

### Comprehensive Clustering Analysis

**Figure 4:**
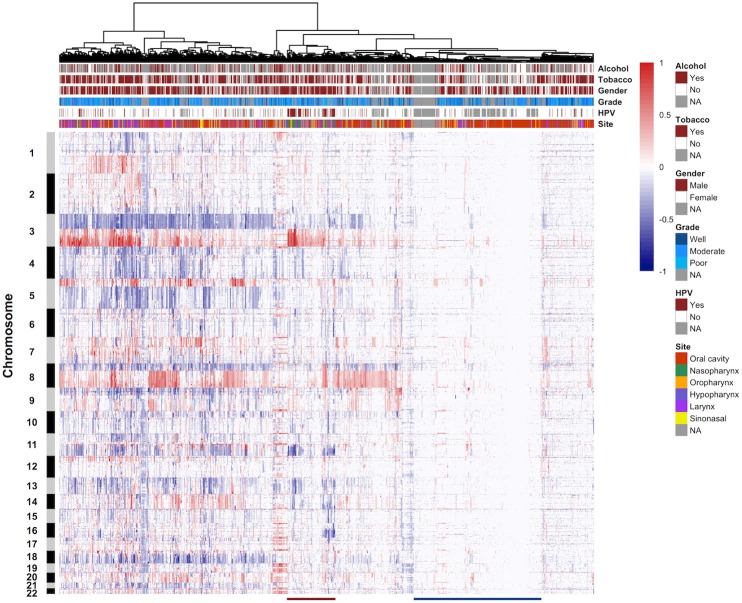
Unsupervised clustering of DNA copy number alteration data. The 1,395 HNSCC samples are arranged along the x-axis and ordered according to their copy number profiles. Chromosome numbers are indicated along the y-axis. Key clinical parameters associated with the samples are displayed in the column header. Red indicates copy number gain, and blue indicates copy number loss. The red line at the bottom of the figure represents a sub-cluster containing a large proportion of HPV-positive samples. The blue line at the bottom of the figure shows a sub-cluster consisted of samples harboring few copy number alterations. NA, not available.

We performed unsupervised hierarchical clustering on the copy number profiles of all HNSCC samples. CNA data were clustered in R based on Euclidean distance using Ward's method, resulting in two major clusters (Figure 4, Supplementary Figure 4). Tumors that were classified in cluster 1 showed a high proportion of the genome affected by CNAs, especially the arm-level events. The most frequent arm-level copy number changes in HNSCC, such as 3q, 8q gain, and 3p, 8p loss, can be found in this cluster. Cluster 2 was characterized by reduced CNAs and tumors were more affected by small focal alterations rather than arm-level events. Furthermore, several additional sub-clusters can be distinguished within both major clusters. For instance, a sub-cluster within cluster 2 was represented by samples harboring few CNAs (M class), a pattern that has been described in previous studies. These genome copy number stable tumors may be primarily driven by mutation rather than CNA. Notably, the hierarchical clustering analysis identified a sub-cluster predominantly composed of nasopharynx tumors and presented a large proportion of HPV-positive samples (Supplementary Table 7). These tumor samples demonstrated a distinctive copy number alteration pattern of 3q gain and lack of 3p loss. These different CNA patterns may be used as markers to cluster HNSCC samples into distinct tumor subtypes, and provide valuable insights into the different molecular mechanisms that underlie HNSCC development.

## Discussion

The high degree of genomic heterogeneity of HNSCC underscores a challenge to distinguish between driver and passenger alterations from aberrant regions. A large number of tumor samples need to be examined in order to identify driver genes that are not detected when each tumor is analyzed individually. In this study, we characterized genome-wide DNA copy number alterations in HNSCC based on published high resolution aCGH and SNP array data. From the overall CNA profile of 1,395 HNSCC samples, we obtained the most frequent arm-level alterations. Previous studies have indicated that broad and focal copy number alterations may represent different characteristics of cancer cells with independent mechanisms (12, 59). While broad copy number alterations may contribute to immune suppression, focal alterations may activate oncogenes and inactivate tumor suppressors that provide tumor cells with a proliferative advantage (60).

We applied GISTIC algorithm (36) to perform focal CNA analysis and identified a number of candidate genes, including driver genes from other tumor types, as well as genes not previously reported to be associated with any cancer. Over-expression of PTPN1 has been linked to colon cancer and breast cancer development and progression (54, 55). The phosphatase activity of PTPN1 is responsible for the regulation of cell motility and invasion, and may enhance the expression of EGFR. High expression of PTPN1 is strongly associated with poor prognosis in gastric cancer (56). These results support the hypothesis that amplification of PTPN1 is important in the pathogenesis of HNSCC. Another promising candidate gene, IRAK1BP1, can activate NF-kappa-B through the TNFR signaling pathway and acts by enhancing RELA transcriptional activity. This gene is associated with inflammation and several autoimmune diseases (61), but not cancer. Our results indicated that IRAK1BP1 may be important in HNSCC development and involved in immune surveillance.

The single catastrophic event chromothripsis is a novel phenomenon of genomic instability and is distinct from the progressive accumulation of mutations model of cancer development (19, 23). The prevalence of chromothripsis is heterogeneous across various cancer types. According to previous studies, chromothripsis on average can be found in approximately 5% of all cancers, with up to 25% in bone tumors (19, 22, 58, 62). We detected 92 chromothripsis cases out of 1,395 HNSCC samples, giving an incidence about 6%. While the underlying mechanisms resulting chromothripsis are still largely unknown, several hypotheses have been proposed, including micronuclei formation (63, 64), abortive apoptosis (65, 66), premature chromosome condensation (67), breakage-fusion-bridge cycles (68–70), and telomere dysfunction (71). The patterns of chromosomal pulverization may reveal underlying mechanisms of chromothripsis in carcinogenesis. In our cohort, 89% chromothripsis cases affect only a single chromosome, and about 3% cases affect 3 chromosomes simultaneously. Chromothripsis involving multiple chromosomes can result from several chromosomes in a micronucleus or is the consequence of a process of aborted apoptosis. It is likely that more than one mechanism contributes to the generation of chromothripsis in HNSCC. Since chromothripsis was more frequently detected in particular genomic regions, the chromosomal pulverization hotspots may contain critical genes for the regulation of cell cycle control, DNA damage, proliferation and apoptosis. For instance, Rausch et al. (24) have associated chromothripsis with TP53 mutations in subsets of medulloblastoma. In our analysis, the most frequently affected chromosome regions were 8p and 11q (account for 18 and 14% chromothripsis cases, respectively), suggesting that these loci were chromothripsis hotspots. The affected genes in these regions may be crucial for chromothripsis formation in HNSCC.

Genomic rearrangement breakpoints may disrupt tumor suppressor genes or create novel gene fusions with oncogenic potential. We performed genome-wide analysis and identified 323 recurrent breakpoints that were more clustered than would be expected if location of CNAs were randomly distributed throughout the genome. These breakpoint-prone regions revealed HNSCC specific genomic instability regions, by comparing with known common fragile sites and non-fragile regions of human genome. A number of potential driver genes detected by GISTIC were located in these regions. These results expanded our understanding of the impact of genomic rearrangements as a cause of HNSCC.

We performed unsupervised hierarchical clustering analysis based on primary tumor samples from six different sites of head and neck region. The results did not show a clear separation between the samples from different locations, which demonstrated the diversity of genetic alterations in a variety of HNSCC sites, as well as the importance to collect large-scale datasets for analysis. In consistent with previous studies, we observed a sub-cluster with few CNAs, which may represent the M class cancer that predominantly driven by mutations rather than copy number alterations (72). In 2015, the TCGA consortium published a comprehensive systematic evaluation of 279 HNSCCs, and provided an integrated view on the molecular landscape of this cancer type (18). HPV+ and HPV- tumors showed different mutational patterns. In our analysis, most HPV+ tumors displayed specific mutational profiles and were clustered together, suggesting that these tumors reflect a separate genetic subgroup. The distinctive copy number alterations of this group of samples include 3q gain and lack of 3p loss. Human papillomavirus infection has been identified in strong association with oropharyngeal and tonsillar cancers (73–75). However, our cluster analysis showed an enrichment of HPV+ samples in nasopharynx tumors, which was not explicitly noted before. Although the clinical implications of HPV infection in nasopharynx tumors need further investigation, these results are important for patient management and treatment choices.

In summary, we performed a comprehensive characterization of somatic genomic alterations based on a large cohort of HNSCC samples. Several newly identified driver genes in focal alterations, such as IRAK1BP1 and PTPN1, may facilitate molecular mechanism studies of HNSCC. Analysis of chromothripsis regions and breakpoint hotspots directed 8p and 11q as commonly affected regions in HNSCC that may related to disease development. Clustering of patients may provide evidence for patient stratification and differential treatment. These results provide valuable new insights into the mechanisms of genomic instability in HNSCC, and will facilitate the discovery of therapeutic and diagnostic candidates.

## Data Availability Statement

Publicly available datasets were analyzed in this study. This data can be found in TCGA data portal (https://portal.gdc.cancer.gov/) and NCBI GEO database (https://www.ncbi.nlm.nih.gov/geo/). We integrated the following datasets: TCGA-HNSCC, GSE11938, GSE20306, GSE20939, GSE23831, GSE25103, GSE31984, GSE33229, GSE33983, GSE34507, GSE36790, GSE39367, GSE40777,GSE47443, GSE51265, GSE57201, GSE66136, GSE68717, and GSE85514.

## Author Contributions

HC conceived the study. JY and YC collected raw data and performed data analysis. HL participated in interpretation of results and prepared figures. HC and JY drafted the manuscript. All authors read and approved the final manuscript.

### Conflict of Interest

The authors declare that the research was conducted in the absence of any commercial or financial relationships that could be construed as a potential conflict of interest.
