# Supplementary material for: The Landscape of Somatic Copy Number Alterations in Head and Neck Squamous Cell Carcinoma
Source: Front Oncol. 2020 Mar 12;10:321. doi: 10.3389/fonc.2020.00321 (PMC7080958; doi:10.3389/fonc.2020.00321)
Supplement: Supplementary file 8 [file Data_Sheet_1.docx]

Supplementary Material

## Supplementary Figures


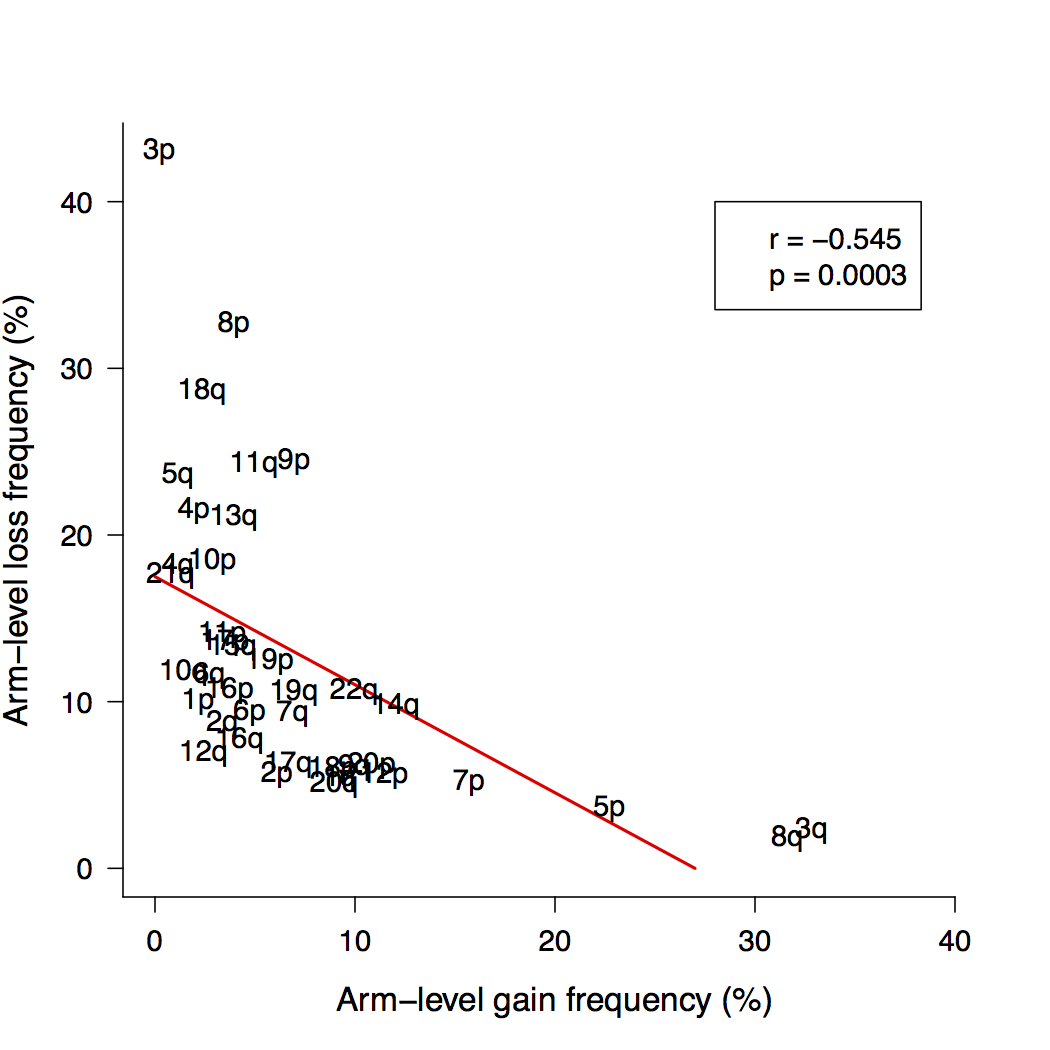


**Supplementary Figure 1.** The frequency of arm-level copy number alterations. The red line represents fitted least squares regression line.


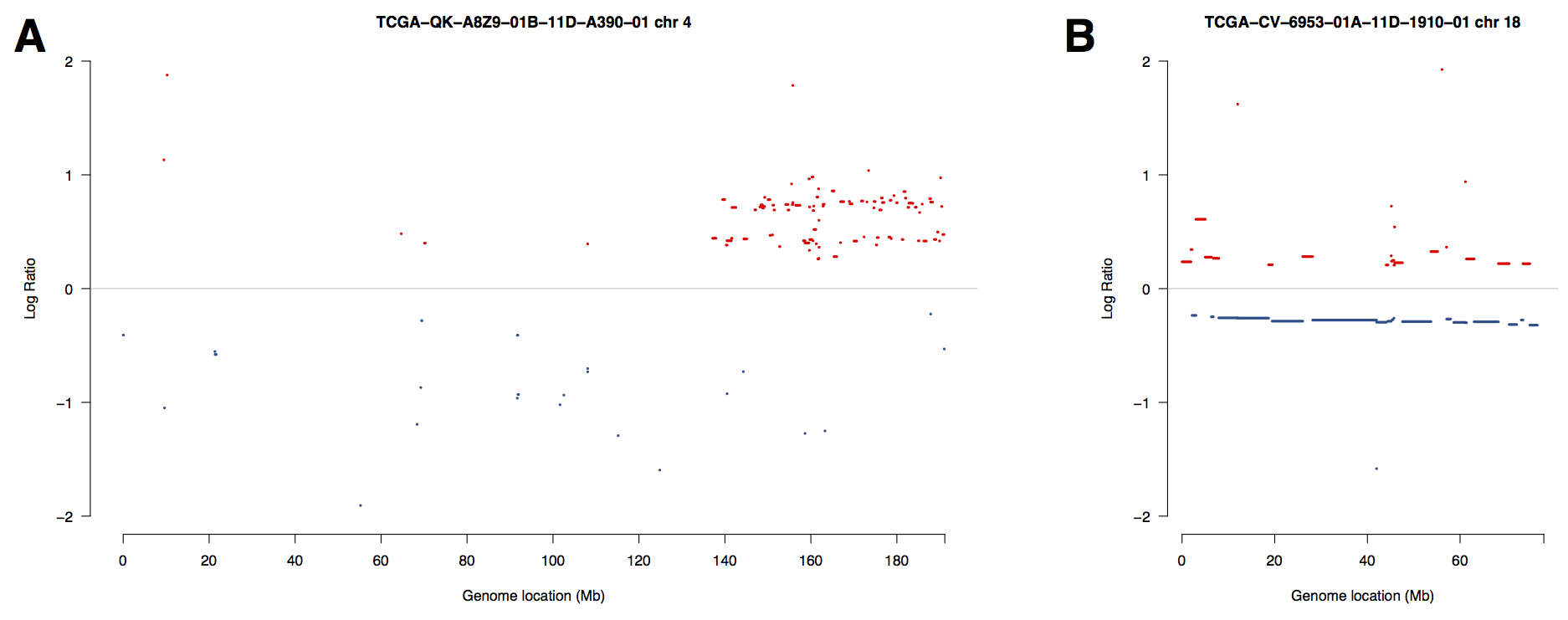


**Supplementary Figure 2.** Examples of localized and chromosome-level chromothripsis. (A) Localized pulverization of chromosome 4. (B) Whole chromosome 18 pulverization. The x-axis indicates genomic locations in Mb, and the y-axis is the log2 value of probe signal intensity. Red and blue lines represent genomic gains and losses respectively.


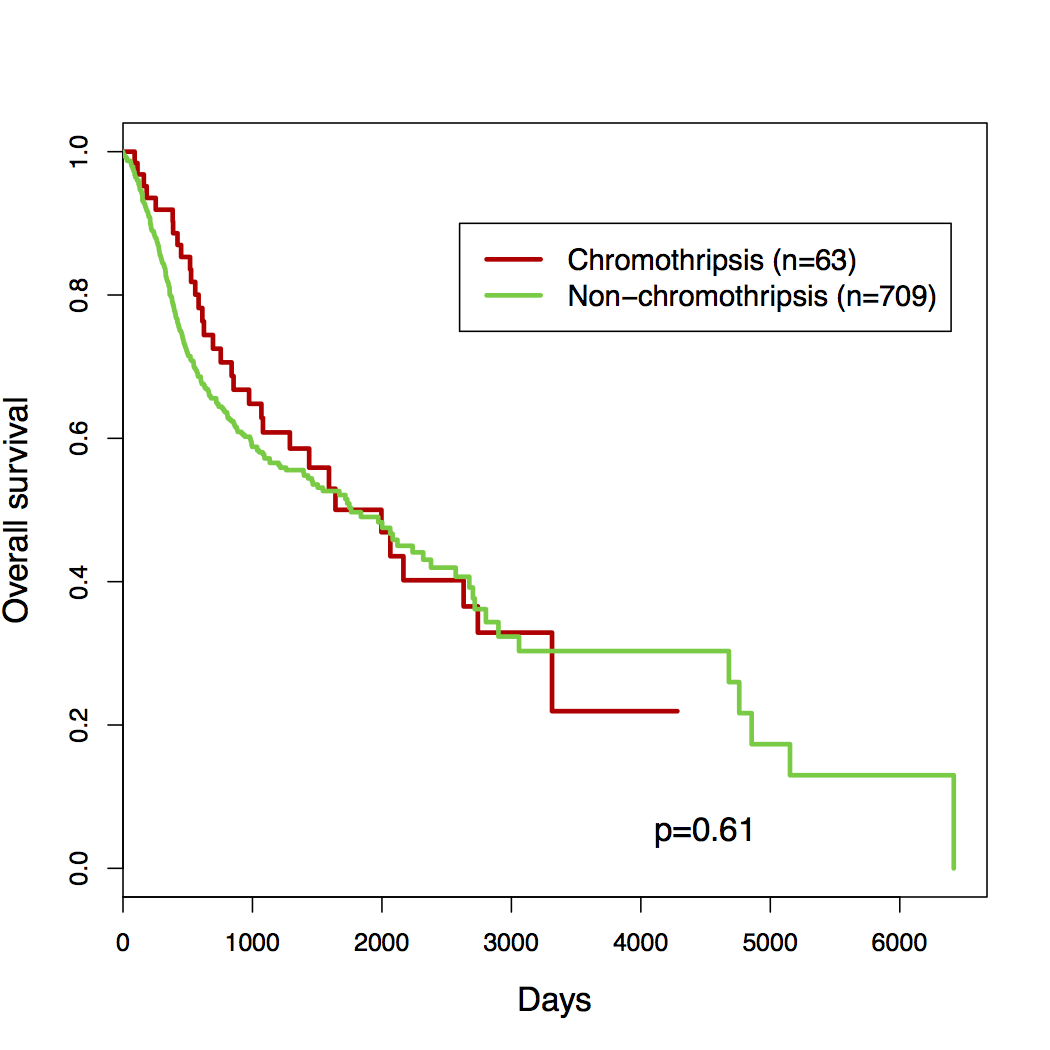


**Supplementary Figure 3.** Kaplan-Meier survival curves for patients with and without chromothripsis patterns. The p value was based on the log-rank test.


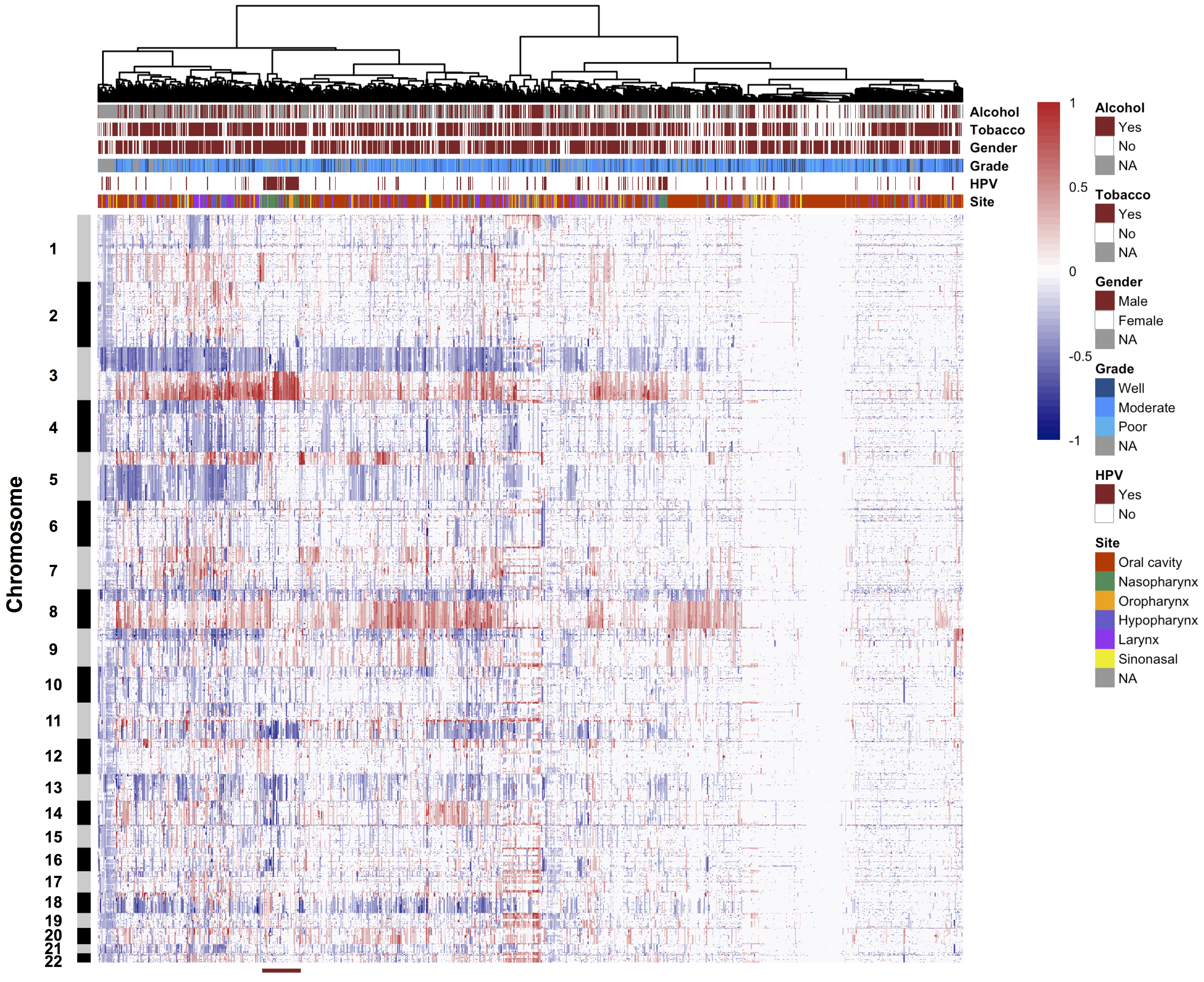


**Supplementary Figure 4.** **Unsupervised clustering of DNA copy number alteration data for samples with known HPV status.** The HPV status of the 1065 HNSCC samples are known. The samples are arranged along the x-axis and ordered according to their copy number profiles. Chromosome numbers are indicated along the y-axis. Key clinical parameters associated with the samples are displayed in the column header. Red indicates copy number gain, and blue indicates copy number loss. The red line at the bottom of the figure represents a sub-cluster containing a large proportion of HPV-positive samples. NA, not available.
